# Underreporting of Viral Encephalitis and Viral Meningitis, Ireland, 2005–2008

**DOI:** 10.3201/eid1909.130201

**Published:** 2013-09

**Authors:** Tara A. Kelly, Piaras O’Lorcain, Joanne Moran, Patricia Garvey, Paul McKeown, Jeff Connell, Suzanne Cotter

**Affiliations:** Health Service Executive–Health Protection Surveillance Centre, Dublin, Ireland (T.A. Kelly, P. O’Lorcain, J. Moran, P. Garvey, P. McKeown, S. Cotter);; National Virus Reference Laboratory, Belfield, Dublin (J. Moran, J. Connell)

**Keywords:** Viral encephalitis, viral meningitis, aseptic meningitis, viruses, herpesviruses, varicella-zoster, enterovirus, ICD-10, surveillance, disease notification, hospitalization

## Abstract

Viral encephalitis (VE) and viral meningitis (VM) have been notifiable infectious diseases under surveillance in the Republic of Ireland since 1981. Laboratories have reported confirmed cases by detection of viral nucleic acid in cerebrospinal fluid since 2004. To determine the prevalence of these diseases in Ireland during 2005–2008, we analyzed 3 data sources: Hospital In-patient Enquiry data (from hospitalized following patients discharge) accessed through Health Intelligence Ireland, laboratory confirmations from the National Virus Reference Laboratory, and events from the Computerised Infectious Disease Reporting surveillance system. We found that the national surveillance system underestimates the incidence of these diseases in Ireland with a 10-fold higher VE hospitalization rate and 3-fold higher VM hospitalization rate than the reporting rate. Herpesviruses were responsible for most specified VE and enteroviruses for most specified VM from all 3 sources. Recommendations from this study have been implemented to improve the surveillance of these diseases in Ireland.

Encephalitis and meningitis are serious inflammatory diseases of the brain that require hospitalization for many patients and are a substantial cause of illness. Although the etiologic agent is not identified for most cases (*1*), viral infection has been reported as a major cause (*2*).

Acute encephalitis is characterized by a triad of fever, headache, and altered mental status (*3*). Common features include disorientation/depressed level of consciousness; disturbances of behavior, speech, or executive function; and diffuse or focal neurologic signs such as cranial nerve dysfunction, hemiparesis, or seizures (*3*). Capillary and endothelial inflammation of cortical vessels is a striking pathologic finding, occurring primarily in the gray matter or the gray–white junction (*4*). These features distinguish encephalitis from the more commonly encountered meningitis. The most common agents that cause acute viral encephalitis (VE) are herpes simplex virus (HSV) and varicella-zoster virus (VZV) (*5*).

A distinction must be made between acute VE and autoimmune/postinfectious encephalitis, which can occur with a variable latent phase between acute illness and the onset of neurologic symptoms (*6*,*7*). This distinction is critical because the management and prognosis are often quite different (*4*). Evaluation of cerebrospinal fluid (CSF) following lumbar puncture is essential for accurately diagnosing disease, unless its collection is contraindicated because of high intracranial pressure (*4*). In this study, we did not attempt to ascertain the prevalence of autoimmune/postinfectious encephalitis in Ireland.

Aseptic meningitis refers to a disease with acute onset of symptoms and obvious signs of meningeal involvement, in which an etiologic agent is not apparent after bacterial culture of CSF (*8*). The disease is often associated with lymphocytic pleocytosis without other cause. These patients usually lack altered sensorium or abnormal global or focal neurologic signs (*3*,*4*). Viruses are most commonly associated with these clinical manifestations, most frequently, enteroviruses, herpesviruses, and arboviruses (*9*). Under the 2003 case definitions covering this study period, laboratory evidence involving CSF analysis or immune response, in addition to clinical diagnosis, was necessary for the reporting of VE and VM to public health departments (*10**,**11*).

In this study, we examined 3 different available data sources to estimate how well data reported to public health authorities and captured by the Computerised Infectious Disease Reporting (CIDR) passive surveillance system, during 2005–2008, reflected the incidence of VE and VM in Ireland. CIDR is the real-time Internet-based surveillance system for 93.9% of all notifiable infectious diseases reportable by statute in Ireland. We compared cases reported to CIDR with laboratory detection of cases from the National Virus Reference Laboratory (NVRL) and cases identified from hospitalized patient discharge information in the Hospital In-Patient Enquiry (HIPE) scheme. In 2005, eight (24%) of the 34 public hospital laboratories, in addition to the national reference laboratories, were connected directly to CIDR. This connection increased to 16 (47%) in 2008. Hospital/community physicians who manage VE or VM patients using laboratories which were not directly connected to CIDR, were obligated to report notifiable diseases to CIDR through the local department of public health.

The data sources and the process of reporting notifiable diseases in Ireland are shown in Figure 1. HIPE is an active system that monitors hospital activity and is independent of either NVRL or CIDR. The diagnoses and procedures recorded on the patient’s chart are coded by hospital administration staff according to the International Classification of Diseases and Related Health Problems, 10th Revision (ICD-10). A copy of this database, which includes only publicly funded hospitals, was accessed by using Health Intelligence Ireland (HII), an open source software program developed by the Irish Health Services Executive (www.healthatlasireland.ie/f1live).

**Figure 1:**
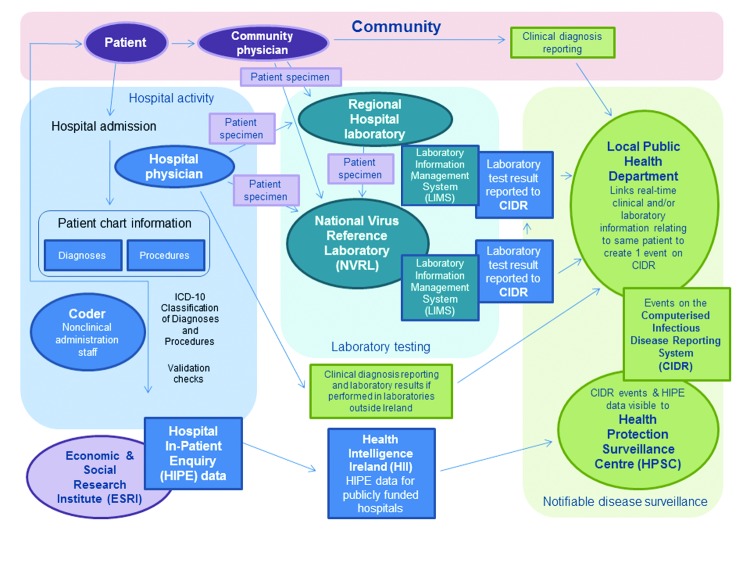
Sources of information of clinical and laboratory diagnoses of notifiable infectious diseases in Ireland and the routes of reporting such diagnoses to the national Computerised Infectious Disease Reporting system.

A previous study suggested an underestimation of the incidence of acute encephalitis and VM in the northeastern part of Ireland (*12*,*13*). In that study only hospital activity and CIDR data were compared, and no distinction was made between laboratory or clinical criteria for diagnosis of these diseases at that time.

## Materials and Methods

### CIDR Events

Events are composed of >1 clinical diagnoses and/or laboratory test results for a single patient (Figure 1). We extracted recorded events of VE and VM from CIDR. The organism responsible for each disease is captured as enhanced surveillance.

### Hospitalizations

HIPE collects information regarding in-patient and day-case hospital activity. Each HIPE discharge record represents 1 episode of care. Patients may have been admitted to hospital(s) more than once with the same or different diagnoses. A HIPE discharge record is generated after a patient is discharged from, or dies, in the hospital (*14*). The record accessed through HII contains an anonymized medical record number. Duplicate discharges for the same anonymized medical record number were found by HII and removed.

Hospital discharges from January 1, 2005, through December 31, 2008, coded with the ICD-10-AM (Australian Modification), fourth edition diagnosis codes A83, A84, A85, A86, B00.4, B01.1, B02.0, B05.0, and B26.2 were defined as VE. Discharges associated with the codes A87, B00.3, B01.0, B02.1, B05.1, and B26.1 were defined as VM. We determined the proportion of these codes recorded in any of the diagnosis fields and in the primary diagnosis field only.

Hospitalizations in which a lumbar puncture (ICD-10-AM code 39000–00) was listed were extracted from HIPE for comparison with the number of CSF samples received at NVRL for virologic testing and with those in which a virus was detected from patients with symptoms of acute encephalitis or meningitis. Deaths were determined by searching for patients on HIPE who “died with post mortem” or “died no post mortem.”

### Calculations

Denominator data for population-based incidence rates (of CIDR events, laboratory confirmations, and patient discharges) correspond to published Irish Census 2006 Principle Demographic Results (n = 4,239,848; Central Statistics Office, 2007; www.cso.ie/en/statistics/population). To prevent the identification of individual hospital in-patients, when 5 or fewer discharges or patients for a particular diagnosis were found, we depicted the number as ≤5.

The statistical significance of the difference in proportions was tested by using the χ^2^ test or Fisher exact test as appropriate (Stata 11.1; http://www.stata.com/stata11/point1.html); 95% CIs were also calculated. Male-to-female ratios (MFRs) are presented as the number of male patients to 1 female patient.

### Investigation of CSF Specimens at NVRL

Investigation of CNS infection is primarily based on detection of HSV-1 DNA, HSV-2 DNA, or VZV DNA. When appropriate, on the basis of clinical manifestations/underlying clinical issues and in collaboration with the clinical teams managing a patient’s condition, testing can be performed for human herpesvirus 6 DNA, Epstein-Barr virus DNA, cytomegalovirus DNA, JC virus DNA, enterovirus RNA, enterovirus 71 RNA, measles virus RNA, mumps virus RNA, and lymphocytic choriomeningitis virus RNA. When encephalitis or meningitis is suspected because of arboviral infection, the infection is diagnosed by detecting IgM in serum, CSF, or both. On the basis of the patients’ clinical and travel history, tests were performed for serologic evidence of the following: West Nile virus, Venezuelan equine encephalitis virus, Japanese encephalitis virus, yellow fever virus, dengue virus, eastern equine encephalitis virus, western equine encephalitis virus, St. Louis encephalitis virus, Powassan virus, La Crosse virus, and tick-borne encephalitis virus.

In addition to the investigation of CSF, molecular analysis and culture of fecal samples were also performed. The detection of enterovirus provides additional circumstantial evidence for the viral etiology of the CNS manifestations.

NVRL does not routinely receive convalescent-phase serum samples; therefore, investigations of acute- or convalescent-phase serum are uncommon. Intrathecal antibody testing is not performed at NVRL. On the rare occasion when such specimens are sent to an international reference laboratory, the results of such testing would be known to NVRL if the specimens were originally sent through NVRL to that laboratory.

Herpesvirus DNA is often not detectable in CSF from encephalitis/meningitis patients. It can nonetheless be confirmed through evidence of elevated specific intrathecal IgG. The absence of such testing in our analysis may have led to underdetection of meningitis attributable to herpes group viruses, particularly VZV, which other studies have found to be the most common viral cause for VM, including a 2001 study in Finland (*15*).

We acknowledge that molecular investigation may not always detect DNA or RNA soon after onset of symptoms or following antiviral treatment. However, collaboration with the clinical teams dealing with the patient can often highlight whether this could be an issue. Results were extracted from the laboratory information management system (LIMS) WinPath (CliniSys Solutions Ltd, Chertsey, UK) and configured for upload to CIDR.

## Results

### Reporting to CIDR

**Figure 2:**
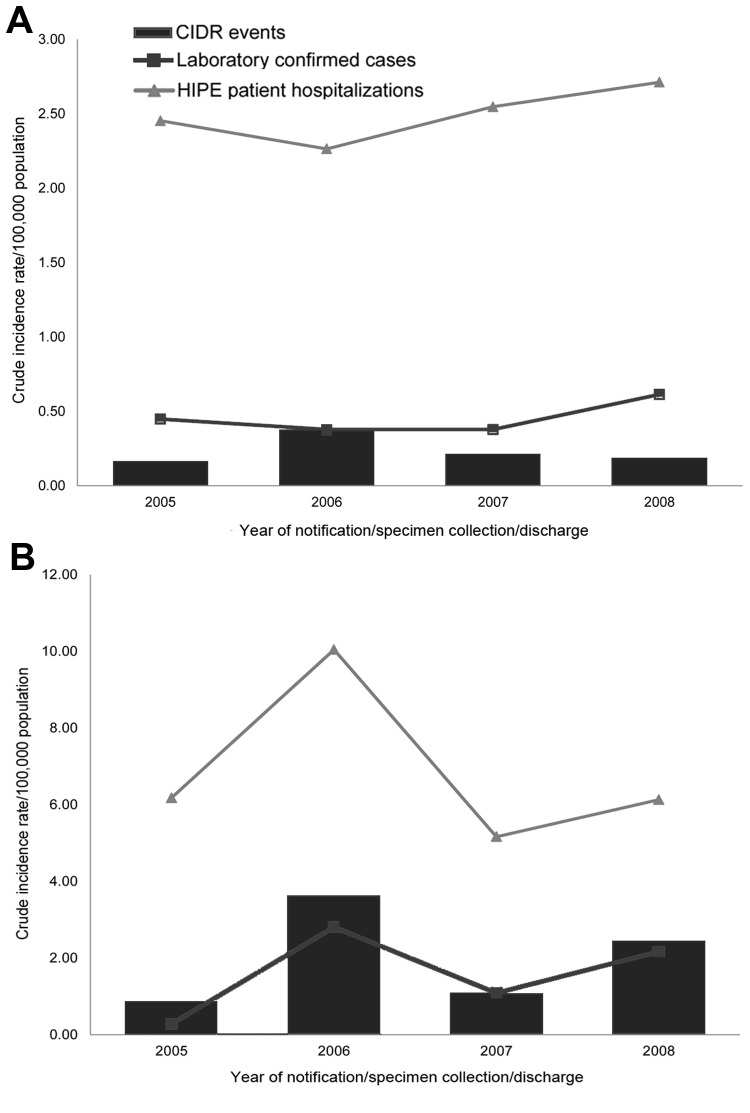
A) Crude incidence rates per 100,000 population of viral encephalitis events (Computerised Infectious Disease Reporting system [CIDR]), hospitalizations (Hospital In-Patient Enquiry [HIPE] patients), and laboratory-confirmed cases (National Virus Reference Laboratory [NVRL]), Ireland, 2005–2008. B) Crude incidence rates per 100,000 population of viral meningitis events (CIDR), hospitalizations (HIPE patients), and laboratory-confirmed cases (NVRL).

Between 2005 and 2008, a total of 40 VE events and 341 VM events were reported to CIDR (Table 1). Of these, 39 VE events and 261 VM events were classified as confirmed. The rates of VE and VM by data source and year are shown (Figure 2, panels A, B) with the highest rates of VM occurring in 2006.

**Table 1:** Viral encephalitis and viral meningitis CIDR events, laboratory-confirmed cases, and patient hospitalizations by causative virus, Ireland, 2005 to 2008*

ICD code	Description of code	No. CIDR events	No. NVRL confirmed cases	HIPE data						
				No. discharges	No. patients	No. deaths	Case-fatality ratio (%)	Total no. bed days	Mean no. bed days	Mean no. ICU days
A83	Mosquito-borne encephalitis	–	–	≤5	≤5	0	0.0	≤5	≤5	0.0
A84	Tick-borne encephalitis	–	–	≤5	≤5	≤5	50.0	91	45.5	26.0
A85	Other viral encephalitis	–	–	42	25	≤5	4.0	512	12.2	1.2
A86	Unspecified viral encephalitis	4	–	283	223	10	4.5	4,112	14.5	1.2
B00.4	Herpesvirus encephalitis	17†	42‡	195	95	11	11.6	2,857	14.7	0.9
B01.1	Varicella encephalitis	12§	35	28	28	≤5	7.1	706	25.2	2.3
B02.0	Zoster encephalitis			43	43	8	18.6	789	18.4	1.5
B05.0	Measles encephalitis	–	–	–	–	–	–	–	–	–
B26.2	Mumps encephalitis	5	–	≤5	≤5	0	0.0	18	18.0	3.0
	Enteroviral encephalitis	2	–	–	–	–	–	–	–	–
Total viral encephalitis		40	77	595	418	33	7.9%	9,086	15.3	1.2
A87.0	Enteroviral /coxsackievirus /echovirus meningitis	210¶	215#	60	52	0	0.0	428	7.1	0.8
A87.1	Adenoviral meningitis	–	–	≤5	≤5	0	0.0	25	5.0	0.6
A87.2	Lymphocytic choriomeningitis/lymphocytic meningoencephalitis	–	–	57	47	≤5	6.3	886	15.5	0.1
A87.8	Other viral meningitis	–	–	36	34	0	0.0	267	7.4	0.2
A87.9	Viral meningitis unspecified	91	–	1,048	990	≤5	0.1	5,701	5.4	0.1
B00.3	Herpesvirus meningitis	12**	32††	14	14	0	0.0	365	26.07	4
B01.0	Varicella meningitis	11‡‡	22	11	10	0	0.0	80	7.3	0.0
B02.1	Zoster meningitis			13	12	0	0.0	231	17.8	0.1
B05.1	Measles meningitis	–	–	≤5	≤5	0	0.0	6	6.0	0.0
B26.1	Mumps meningitis	16	–	≤5	≤5	0	0.0	22	4.4	0.0
	Parechovirus meningitis	1	–	–	–	–	–	–	–	–
Total no. viral meningitis cases		341	269	1,250	1170	≤5	0.3%	8,011	6.4	0.2

*CIDR, Computerised Infectious Disease Reporting system; ICD, International Classification of Diseases, 10th Revision; NVRL, National Virus Reference Laboratory; HIPE, Hospital In-Patient Enquiry; ICU, intensive care unit. †Includes 14 herpes simplex virus (HSV), untyped; 2 HSV-1 and 1 human herpesvirus type 6 events.  ‡Includes 37 HSV-1, 1 HSV-2, and 4 human herpesvirus type 6 detections.  §Includes 11 varicella-zoster and 1 varicella species events.  ¶Includes 205 enterovirus, 1 coxsackie A virus, 1 coxsackie B virus, 2 echovirus, and 1 echovirus type 6 events.  #Includes 211 enterovirus, 3 coxsackie B3 virus, and 1 coxsackie B5 virus detections. **Includes 10 HSV untyped, 1 HSV-1, and 1 HSV-2 events.  ††Includes 7 HSV 1, 5 HSV 2 and 20 human herpesvirus type 6 events.  ‡‡Includes 10 VZV and 1 varicella species detections.

**Figure 3:**
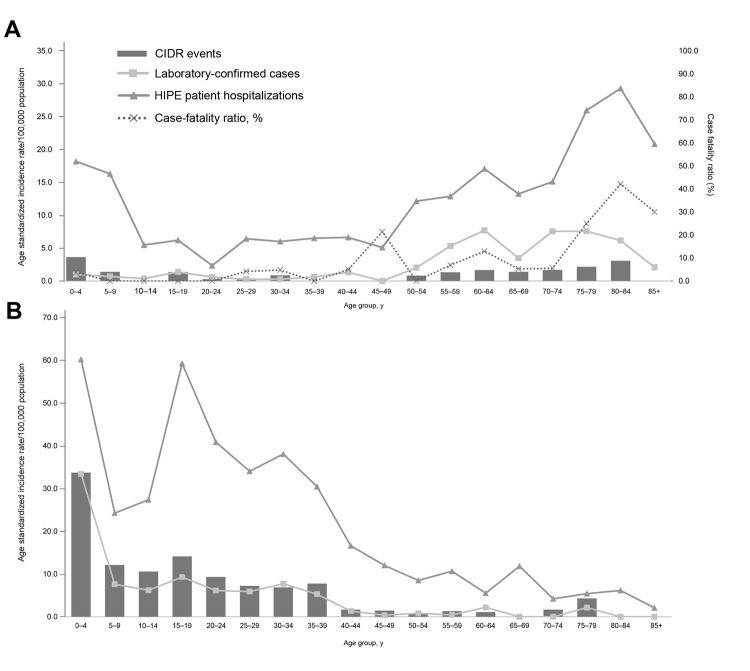
A) Viral encephalitis age-specific incidence rates of events (Computerised Infectious Disease Reporting system [CIDR]), hospitalizations (Hospital In-Patient Enquiry [HIPE] patients), and laboratory-confirmed cases (National Virus Reference Laboratory [NVRL]) by age group, Ireland, 2005–2008. B) Viral meningitis age-specific incidence rates of events (CIDR), hospitalizations (HIPE), and laboratory-confirmed cases (NVRL) by age group, Ireland, 2005–2008. The figure excludes 4 CIDR events and 1 laboratory-confirmed case with patient age unknown.

VE (62.5%) and VM (58.7%) occurred more frequently in male patients (MFRs, 1.67 and 1.42, respectively). The greatest age-standardized incidence rates (ASIR) of VE (27.5%) and VM (29.9%) were for children <4 years of age (Figure 3, panels A, B). For VM, 24.6% of all events involved children <1 year of age.

The highest number of VE events were caused by HSV (40.0%), followed by VZV (27.5%) and mumps virus (12.5%) as shown in Table 1. Only 12.5% of herpesviral events had the type recorded, as opposed to 95% in 2011 (when all laboratories had live links to CIDR, unpub. data). Most VM events were caused by enteroviruses (61.9%, which included coxsackie A and B viruses, enterovirus, echovirus and echovirus type 6), mumps (4.7%), and HSV (3.5%).

**Figure 4:**
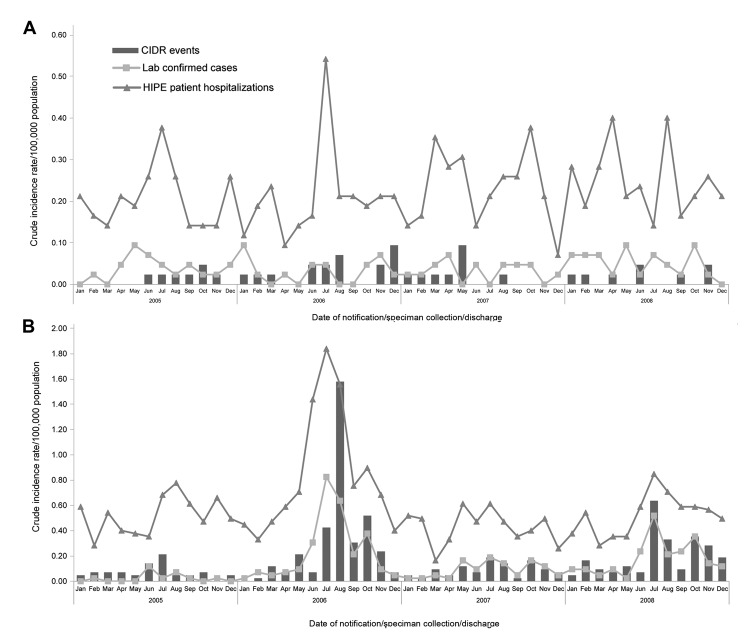
A) Viral encephalitis crude incidence rates of events (Computerised Infectious Disease Reporting system [CIDR]), laboratory-confirmed cases (National Virus Reference Laboratory [NVRL]), and patient hospitalizations (Hospital In-Patient Enquiry [HIPE]), by month and year, Ireland, 2005–2008. B) Viral meningitis crude incidence rates of events (CIDR), laboratory-confirmed cases (NVRL), and patient hospitalizations (HIPE), by month and year.

No deaths from either VE or VM were reported to CIDR. VM reporting was greatest in the month of August; overall rates were highest in August 2006 (Figure 4, panels A, B). Two outbreaks of enteroviral meningitis were reported to CIDR; one in July 2006 (which began in late June) and one in July 2008. No outbreaks of VE were reported in these years.

### HIPE Hospitalizations

During 2005–2008, a total of 418 patients were discharged on 595 occasions with a diagnosis of VE, giving a mean annual patient hospitalization rate of 2.49/100,000 population (95% CI 2.31–2.68) and a mean annual discharge rate of 3.51/100,000 population (95% CI 2.15–4.87); Figure 2, panels A, B). There were more hospital discharges for VM; 1,116 patients were discharged on 1,250 occasions, which corresponds to a mean annual patient hospitalization rate of 6.88/100,000 population (95% CI 4.76–9.00) and a mean annual discharge rate of 7.37/100,000 population (95% CI 5.17–9.17).

A total of 83.0% of discharges of patients with VE and 92.2% of those with VM were considered primary diagnosis discharges. The mean length of stay for a patient discharged with VE was 15.3 days (range 1–254 days; median 7 days), and mean length of stay in an intensive care unit was 1.2 days (range 0–55 days; median 0 days). The average cost per discharge for VE was €9,783.31. For those with VM, the mean length of stay was 6.4 days (range 1–128 days; median 5 days), the mean length of stay in an intensive care unit was 0.2 days (range 0–56 days; median 0 days), and the average cost per discharge was €4,612.77.

Male patients accounted for 53.4% of all VE patients and 53.0% of VM patients (MFRs of 1.24 and 1.13, respectively). The ASIR of patients hospitalized with VE was highest among elderly (80–84 years of age) and very young patients (0–4 years of age; Figure 3, panel A). The ASIR for VM was highest among children aged 0–4 years, followed closely by adolescents ages 15–19 years (Figure 3, panel B).

For the majority (53.3%) of persons hospitalized with VE, viral cause was unknown (Table 1). When an agent was specified (n = 195), herpesvirus was the causative agent of 48.7%, zoster of 22.1%, other (including enteroviral, adenoviral, arthropod-borne, mosquito-borne, and tick-borne viruses) of 14.9%, and varicella of 14.4% of patient hospitalizations. For most (84.6%) persons hospitalized with VM, cause was also unspecified, and when a virus was specified (n = 180), enteroviruses (including coxsackie B virus and echovirus) were the most common etiologic agents at 28.9%, followed by lymphocytic choriomeningitis virus at 26.1% and other at 18.9%.

Thirty-three hospitalized patients with a diagnosis of VE died, giving a case-fatality ratio (CFR) of 7.9% (Table 1; Figure 3, panel A); CFR was highest among those80–84 years of age. Eleven of the 33 VE deaths were associated with herpesvirus encephalitis (CFR 2.6%) and 8 with zoster encephalitis (CFR 1.9%). Ten deaths were from unspecified cause, and the remaining 4 deaths were caused by varicella, tick-borne encephalitis, or other encephalitis-causing viruses. In contrast, VM resulted in fewer (≤5) deaths (Table 1), which were either caused by lymphocytic choriomeningitis virus or unspecified.

A peak of both VE and VM hospitalizations occurred in the summer months, most notably in July and August (Figure 4, panels A, B). For both diseases, the highest number of hospitalizations occurred in July 2006. Of the patients hospitalized for VE, 52.61% had a lumbar puncture, compared with 73.98% of patients hospitalized for VM (Table 2).

**Table 2:** Crude incidence rates per 100,000 population and proportions of lumbar puncture procedures among hospitalized patients with a diagnosis of VE or VM, Ireland, 2005–2008*

Rate or proportion	Year of discharge/laboratory confirmation				Average annual rate or proportion
	2005	2006	2007	2008	
Hospitalization rate for all patients with lumbar puncture†	99.20	116.30	120.05	129.53	116.27
VE patient hospitalization rate	2.45	2.26	2.55	2.71	2.49
Hospitalization rate among VE patients with lumbar puncture	1.18	1.18	1.44	1.44	1.31
% VE hospitalizations with lumbar puncture	48.16	52.21	56.47	53.14	52.61
VM patient hospitalization rate	6.18	10.05	5.17	6.13	6.88
Hospitalization rate among VM patients with lumbar puncture	4.34	7.38	3.89	4.74	5.09
% VM hospitalizations with lumbar puncture	70.23	73.43	75.24	77.32	73.98
CSF collection rate of samples sent for virologic testing	31.46	40.71	39.65	41.53	38.34
Laboratory CSF confirmation rate of VE	0.50	0.42	0.42	0.71	0.51
% Laboratory confirmed CSF for VE	1.59	1.03	1.06	1.71	1.33
Laboratory CSF confirmation rate of VM	0.28	2.83	1.11	2.24	1.62
Laboratory confirmed CSF for VM, %	0.89	6.95	2.80	5.39	4.23
Laboratory CSF confirmation rate of VE or VM	0.78	3.25	1.53	2.95	2.13
% Laboratory confirmed CSF for VE or VM	2.48	7.98	3.69	7.10	5.56

*VE, viral encephalitis; VM, viral meningitis; CSF, cerebrospinal fluid. †All hospitalized patients who underwent lumbar puncture for any reason, diagnostic or therapeutic.

### NVRL CSF Analysis

The NVRL received 6,502 CSF specimens collected during 2005–2008. Of those, 1.3% tested positive for a virus causing encephalitis, and 4.2% tested positive for a virus causing meningitis (Table 2). A listing of causative pathogens of laboratory-confirmed cases is shown in Table 1.

Most CSF specimens indicating VE were from male patients (63.6%; MFR 1.75), as were those indicating VM (56.8%; MFR 1.31). The highest ASIR of laboratory confirmations for viruses causing encephalitis was from patients >55 years of age (Figure 3, panel A) and of viruses causing meningitis were from those 0–4 years of age (Figure 3, panel B). No seasonality was shown in laboratory detections of viruses causing encephalitis (Figure 4, panel A), but a higher number of viruses causing meningitis were detected in the summer months; most were detected in July (Figure 4, panel B).

### Statistical Differences between Data Sources

We analyzed the differences between hospital activity, laboratory confirmations, and events created by using several parameters. When these factors were compared by age of patient, we found a significant difference in the distribution of VE laboratory confirmations and events (Fisher exact test, p<0.001).

Of VE cases with a specified cause, which should have been reported to public health authorities according to the case definitions in use at the time, a significant difference was found in the distribution of VE cases by causative agent between laboratory confirmations and events (Fisher exact test, p<0.001). Lessthan half of laboratory confirmations and 18.5% of hospitalizations were reported. 

Among VM cases for which an organism was specified, a significant difference was found in the distribution of cases by causative agent between hospitalizations and events (χ^2^ = 139.83, p<0.001) and laboratory confirmations and events (χ^2^ = 9.91, p = 0.01). Only 9.2% of patients with illness of unspecified cause were reported. On the other hand, the number of enteroviral events or laboratory confirmations was 4-fold higher than the number of hospitalizations. No deaths had been reported to CIDR, in contrast to the 33 VE deaths and ≤5 VM deaths recorded in HIPE.

## Discussion

During 2005–2008, only 9.6% of cases in hospitalized patients with VE and 29.2% of cases in hospitalized patients with VM were reported to CIDR. Attempting to ascertain the proportion of the difference due to underreporting, we looked at the difference in the percentage of patients hospitalized with a diagnosis for VE and VM and events of these diseases created in CIDR in 2011. By then, 100% of public hospital laboratories were connected to CIDR, which facilitated the reporting mechanism. We found that hospitalized patients reported to CIDR with a diagnosis of VE increased from 9.6% to 20.2% in 2011 and that patients with VM increased from 29.2% to 69.6% (T. Kelly, unpub. data). The laboratories conducting the testing probably contributed to the underreporting. Underreporting or misclassification of diagnoses by hospital physicians without confirmatory tests may also have occurred.

Surveillance data are routinely used to quantify the incidence of disease and to identify outbreaks or emerging viral diseases, and this underidentification of VE and VM in Ireland is of concern. With the emergence of West Nile fever in Italy (*16*), dengue fever in France (*17*) and other European countries, and changes in the mosquito populations of different countries, CIDR must be able to detect possible emerging pathogens.

VE was shown to have greater effects on the Irish health service than VM in terms of deaths, length of hospital stay, repeat hospitalizations, ongoing hospital care, financial costs, and residual damage, although VM caused a greater number of hospital admissions. Similar to the situation in the United States, those seeking treatment for VE and VM were more likely to be male (*9*,*18*). However, a UK study found no difference in VE by sex and showed a lower hospitalization rate for patients with VE (*19*).

VE hospitalizations and events were most prominent in the youngest and oldest age groups. VM followed the same pattern for all 3 data sources (without statistical difference), with peaks in the 0–4 and 15–19 year age groups, which had also been found in Denmark (*20*). The peak in adolescents may reflect a large mumps outbreak that occurred during this period which primarily affected adolescent boys (*21*). The mumps outbreak in this age group may be attributable to a combination of factors, including social and environmental exposures and waning immunity to mumps (*22*). A previous seroepidemiologic study had identified inadequate immunity to measles, mumps, and rubella in school-aged children in Ireland (*23*–*25*).

Similar to results of studies in other countries (*15*,*19*,*26*), herpes and varicella-zoster viruses were the most commonly specified causes of acute aseptic encephalitis. As for VE, most (84.6%) VM hospitalizationshad an unspecified cause, which is similar to the 92% reported in the United States by the Centers for Disease Control and Prevention (*9*), which also reported that the highest rates of specified VM were due to enterovirus infection. The large number of enteroviral meningitis events, which are not reflected in hospital activity, suggests that many enteroviral meningitis cases are classified as unspecified.

We did not identify seasonal trends in VE incidence but did identify a higher incidence of VM during the summer months, as found in other studies (*3*,*9*). Across all 3 data sources, the highest rates of VM were found in July 2006 and July 2008, correlating to periods when community outbreaks of enteroviral meningitis were reported to CIDR. This seasonality for VM continued in subsequent years (*27*).

Because lumbar puncture has a key role to play in the accurate diagnosis of VE and VM, we evaluated the number of patients with VE and VM who had undergone this procedure and found that a higher percentage of patients with suspected VM had undergone lumber puncture than did patients with suspected VE. We also found that a higher percentage of specimens tested positive for a virus causing meningitis. The percentage positive for VE was remarkably low. This finding may reflect either a higher contraindication rate to lumbar puncture in VE patients, a problem confirming the diagnosis of encephalitis, or an increase in the rate of intrahospital transfer of severely ill VE patients to other hospitals with expertise in VE case management (in which case, repeat lumbar punctures would not be usual). It is also possible that alternative noninvasive diagnostic tools were used in patients for whom lumbar punctures were contraindicated, such as magnetic resonance imaging or electroencephalographic testing (*5*).

As a result of this study, new case definitions (*28*) have been implemented that incorporate a case classification for “possible” VE (enabling the reporting of patients meeting clinical criteria without laboratory confirmation). Only 52% of NVRL confirmations were reported to CIDR, but the NVRL Laboratory Information Management System extract used to report positive test results to CIDR was updated to capture omitted results. HIPE coding errors had also been identified in this study, and improved training and data entry validation measures were put in place. Clinical guidelines are being prepared by the Health Protection Surveillance Centre, which aim to improve both the investigation of VE and VM and reporting by physicians. Additional modifications to the list of notifiable diseases (*29*) include new case definitions for dengue fever, West Nile fever, and chikungunya disease, specifically, as well as new case definitions for hospitalized patients with chickenpox caused by VZV. The enhanced surveillance for influenza will now capture encephalitis.

Potential limitations of this study include the presentation of hospitalizations as numbers of patients hospitalized rather than numbers of discharges. Patients obtain a new medical record number when admitted to a different hospital, and patients who are transferred could be counted twice, but the transfer of patients is are considered more likely to affect numbers of VE cases than VM, because of disease severity. We do not have a unique patient identifier in Ireland or a single health information system, as do some Scandinavian countries. HIPE data are collected with the sole purpose of being a source of hospital activity information and, under the Data Protection Act, cannot be used for any other purpose; as such, hospital information cannot be linked with surveillance data (*30*).

This study underscores a key disconnection between health care providers, diagnostic laboratories, and disease reporting. Further investigation of the perspective of attending physicians, laboratory directors, and public health officials may identify approaches for establishing more effective communication between each group on the essential issues of expeditious linking of pathogen identification with clinically apparent disease and reporting to public health. We recommend a follow-up study comparing rates with those when the new case definition (implemented March 2012), improved laboratory reporting, and clinical guidelines have been put in place, to evaluate whether there has been an effect by these changes. Analysis of the referral source of clinical diagnoses and laboratory results to CIDR, specimen type, and laboratory testing performed would facilitate better understanding of the proportion of difference between HIPE and CIDR because of underreporting. Such analysis would also provide feedback and education to the partners involved in health protection as an aid in highlighting the value of the surveillance of these diseases in Ireland and the detection of possible threats to public health.
